# The Diagnosis of Syphilis

**Published:** 1880-09

**Authors:** Fessenden N. Otis

**Affiliations:** Clinical Professor of Veneral Diseases


### THE DIAGNOSIS OF SYPHILIS.

By FESSENDEN N. OTIS, M.D.,
Clinical Professor of Veneral Diseases.

We have one or two cases, gentlemen, that I saw in passing on my way into the lecture room, to seem to make it worth while to consider the value of time as an element of diagnosis in veneral diseases. You know there is a characteristic difference in time between the appearance of the two diseases, chancre and chancroid. Chancroid commences to show a destructive action at once on contact, so that we expect to find positive, well marked evidences of chancroid long before we would have evidence of syphilitic disease. This destructive action commences at the moment of contact. It begins by setting up a necrosis, which first shows itself in a congestion of the parts, then as a perceptible destruction of the part, visible under the microscope before it is perceptible to the naked eye, commencing as a minute point, and then extending by a rapid process of molecular degeneration. The highest type of ulcerative action is this chancroid. We are accustomed to talk about the chancroid having an incubation of three to six, seven, or eight days. This is a very loose way of talking. There is no such thing as an incubation, either in the chancroid or in the chancre, as we shall see as we pass. But in chancroid particularly, there is no reason for calling it an incubation, when it is only because it is so small that your attention is not called to it at first. The sore is there at the commencement; it is very small when it attracts your attention; it may not be until four, six, or ten days. But when a sore appears as an ulcerative lesion within ten days, where we generally find these sores, on the penis, then we say this is a chancroid. We say it is a chancroid, because of the time which has elapsed from the time of contact up to its characteristic development. So, time is a valuable element in making up our minds. Now, if, before we find anything that is characteristic, twenty days, fifteen days, fourteen days, anything over ten days, have passed, we say that is very suspicious; that may be something else. What else? Why, true chancre; syphilitic trouble; the initial lesion of syphilis. And that is a lesion which becomes in a sense ulcerative, as I have explained to you, but has an entirely different nature, ulceration connected with it being one of two varieties, either accidental, as the result of superimposed trouble upon the original condition, which obscures it, and not connected with it except in that sort of way, or caused by a disturbance of the circulation in the part, from the accumulation of material which has been growing at that point. I cannot tell you too often of this difference, which so many have been in error about heretofore; that the one is a process of ulceration, of necrosis *per se*, and the other is a process of proliferation, which results in a lesion from the fact that there is so great proliferation that the tissues are filled up with the cell material, and the vessels of nutrition are choked, and a molecular necrosis is the result. This comes on later. We do not look for the accumulation of cells as a characteristic of the initial lesion of syphilis before the twenty-first day, and then we find it not necessarily as an ulceration, except the ulcerative lesion is not the initial lesion of syphilis, but we find it as a little nodule, a little papule, without any breach of tissue whatever. This is just as important, and may never be anything else. It may pass away without ever having come to an ulcerative point at all. That is the way it begins.

Now, in some cases this very soon breaks down, from a cause that I will tell you of, and there appears an ulcer there. We look at it, and say, well, what is that? Well, when it does appear, if it comes, three, six, eight, ten days after connection, then we say it is chancroid. Whatever else there may be about it, it is chancroid, probably. But after twenty or thirty days have elapsed and you find such a lesion, then you hesitate; you stop to inquire into it; you make the matter of time an important point in your diagnosis.

When did this trouble first come to your notice? "About three days after connection." Had you any connections previous to that? "Yes, sir, about two months previous."

Now, here, you see, is a point of importance, gentlemen; and it is things of this kind that are so necessary to be borne in mind when you come to make a diagnosis and prescribe treatment. A man comes to you and says, "here is a sore which I got from a woman three days ago." Now, perhaps he did and perhaps he did not. There is one thing very certain about it, *he thinks* he did. Now, he has had connection two months before, perhaps, (not referring to the reply of this patient); but two months before he had connection with a woman whom he had full confidence in; he does not think it possible that he could have contracted the disease from her; yet, he may have acquired a syphilis from her, that has gone on in this clean little papular sort of way, not attracting his attention until he had connection with another woman whom he begins to watch, and two or three days afterward he sees this little sore.

It is not well that we accept the simple statement of the patient, that this sore is the result of contact three days before. We must look back, and see whether there may not be something behind that, and that this recent connection has simply called his attention to a trouble, the result of a former connection; that it is really a lesion of syphilis and not a more innocent trouble. We may thus be saved many mistakes which have excused patients in doing things which they would not have done had they known the real nature of their disease.

Now, let us see what you have here. You had no connection at all in the interval between your last connection and the one two months previous? "No, sir." This matter of diagnosis, gentlemen, is one well worth our spending some time upon. Very much mischief has been done by mistaking the initial lesion of syphilis for sores of other varieties, as chancroid, or a simple lesion. It is a very grave matter to overlook syphilis, and give your patient to suppose he has nothing but a simple lesion, and when the simple lesion heals he thinks that is the end of it, and then subsequently his trouble proves to be something else, and perhaps he does not find out until he has infected somebody else.

Now, here we have some little points of ulceration—sharply cut little points; here is a sharply cut loss of tissue over a distinct depression; and here there is another of the same kind which is covered up by a little unhealthy granulation. Now, those of you who wish to come nearer will see there a little chancroid; it is very small; it would not attract attention, perhaps, but for the fact that the chancroid is tender; it has an inflamed border, and one of its characteristics is its sensitiveness. Now, that its characteristic of about the third or fourth day from its first appearance as a little pimple. This little sore here he had not noticed, because it was in a fold of the mucous membrane, and is very small, about the size of half a pearl of barley; and he is getting some more little pimples. Now, here we have this form of ulcerative sore sharply cut, with an inflamed border, and it is very tender; that is one characteristic of it; and then there is another characteristic of it whidh is present here, that there are several little pimples; it is a multiple lesion, which is characteristic of this form of trouble, because the secretion of it is probably setting up the same destructive action that is characteristic of the original sore. Now, the secretion in the initial lesion of syphilis has no such property in itself. The secretion of the initial lesion of syphilis is a bland, transparent fluid, introduced upon the surface of the skin, the mucous membrane. Under the surface of the mucous membrane of the patient it does not set up any irritation whatever. The tissues heal as if nothing of the kind were present. But if you introduce the secretion from this sore under the skin, or lay it upon the mucous membrane so that it shall lay in close contact between the folds for a length of time, by its own property of erosion it will make its way into the tissue, and set up a lesion exactly like that from which it was derived; so that the characteristics of this sore are, in the first place, the action beginning immediately after contact; then its going on as an active destructive process, its having an inflamed border, and its being sensitive to the touch; its having a secretion which is purulent (whereas the one we have just been talking about is serous); and this purulent secretion has the property of contagion, of communicating a similar action to the tissues with which it is brought in contact, and establishing a general sore; and to that property we are indebted for its multiple character, so that when you see half a dozen sores on the penis you say at once, there is chancroidal action. The accumulation of cells which brings about the initial lesion of syphilis does not occur in that way. We have but a single one, as a rule. Sometimes there are more. Where the inoculation takes place at different points we may have several initial lesions of syphilis, but this is rare. Inoculation usually takes place at a single point, and the cells heap up at this point, and finally make themselves manifest by a papule, which may or may not go on to an erosion or apparent ulcerative action.

This young man, then, says that this came on three or four days after connection. You can understand now why it has done so. All that was required was time enough for the infecting material to make its way into the tissue. Now, this length of time varies in different cases. If it happens to be upon the skin of the penis, where it is whole, it may stay there for some time; and if the man is cleanly in his habits it may be washed off and there may be no evidence of it at all, he may not suffer from any trouble whatever; but if it gets in a fold, as you see it here, when the prepuce is drawn back, where the skin naturally falls in folds, it may after a time get up an ulcerative action. It is slow in such a case as that, and you may be deceived. It may be ten or fifteen days, or even longer, possibly, after contact before the sore comes to such a point as to draw your attention to it. But the common place for the occurrence of this sore is in the mucous membrane, the semi-mucous membrane of the reflection of the prepuce as it folds around the frænum, in the little sulci that are formed on either side of the frænum just at the commencement of the fossa glandis. This is the favorite seaf, because here the wrinkles are numerous, the skin is very tender, the sebaceous follicles are numerous, the parts are constantly kept in a moist condition, and this is most favorable for the action of the virus. The consequence is, this is the very common locality for the sore, around the fossa glandis; or it may occur at any point of abrasion, at any wound; and this latter is the way in which we find the sores appearing within a very short time after contact. Perhaps within twenty-four or forty-eight hours there may be evidence of suppurative action where there has been a wound produced and the delay for the virus to work its way through the superficial covering has not been necessary.

This seems to have required three or four days from the time of contact until he noticed this trouble in the fossa glandis, the first one; and the others have come on since. But let us inquire into it thoroughly. When did you have the connection? "About six weeks ago." The connection to which you attributed this? "Yes, sir." And how long after connection did you notice something? "About four days" Was this sore here? "No, sir, it was a running." Then it was not a sore that made its appearance three or four days after connection? "No, sir, it was a running."

Why, we have here a complication, and we are reminded of the fact that we may have several diseases acquired at the same time, at a single connection. He may get a gonorrhœa, he may get a chancroid, he may get an initial lesion of syphilis, all at once. There is no law against it, either pathological or physiological. This sometimes occurs. A single instance of this kind I can recall, where a very amiable young gentleman came down here to visit his brothers, who were used to the ways of the city. He was troubled with seminal emissions, and his brothers thought it would help him to have connection. So they took him out one night, in order to cure him of his seminal emissions, very much against his sense of right. His moral sense was considerably disturbed about it, but he was over-persuaded, and went *just this once,* and after that he could not be persuaded. But three or four days afterward, he began to have a discharge, such as this young man seems to have had, and then three days after that he had some chancroids, and about fifteen or twenty days following one of these chancroids indurated and became an initial lesion of syphilis. Now, that is not uncommon. It is not uncommon for several diseases to be acquired at once. These diseases may coexist, from the fact that one, the gonorrhœa, is a disease of the mucous membrane; it is not assocsated with ulceration; the other, chancroid, is an ulcerative process which comes from contact with a similar ulcer; and the other disease, syphilis, may be engrafted upon any sort of lesion. It is well, perhaps, to say in passing that it is not so likely to take place in active chancroid, and I question very much whether it ever does so except in a way to be mentioned immediately, from the fact that the cell which produces the trouble would be very likely to be destroyed by the active virus. But the way in which it does occur is that the syphilitic cell enters an abrasion, and it does not take a great while for it to get down out of reach of any chancroidal action. In this way, before the chancroidal action is fairly set up, the syphilitic cell has dipped into the lymphatic channels, and deliberately goes on to build up its characteristic accumulation until after the chancroid has healed and got out of the way, when a little nodule is left there which is significant of syphilis.

These things render it somewhat difficult in many cases to make out what disease we have before us, because they are associated with each other, superimposed upon each other in some cases, and consequently it becomes necessary for us to analyze these cases with very great care, and to seek out the landmarks which will enable us to decide positively as to which disease or how many of them are present in any given case.

Now, here, four days after connection, we find this young man complaining of a running. Did that smart you? "No, sir, it only run just the least bit."

Well, now, you see we have constantly to change front in getting over a case like this. We find something which seems to promise one trouble, and looking a little more carefully into it, we find it is something else. I supposed from what he had said, viz: that the trouble followed three or four days afterward, and that it was a running from the penis, we were going to have a case of gonorrhœa from which he recovered; but this seems not to have been the case, for it seems now he had this running lasting a day or two only. If you have a gonorrhœa lasting only that length of time you are surprised at it; three or four weeks is more like the truth. But where it stops like this, it must belong to some other trouble. It may be a little leucorrhœa which starts up, irritating the mucous membrane, and passes off in three or four days; there are plenty of such cases. It may be a little deposit of chancroidal virus within the meatus, out of sight, and here the irritative action goes on, and the first you notice is a little weeping. The man thinks he is going to have a gonorrhœa. But his discharge does not go on and increase from day to day in that sort of way, but his penis is a little bit sore and a little red; perhaps he may notice a little bit of blood, if he has a pretty tight meatus, and if he looks carefully, opening the meatus, he will find a little lesion, a little chancroid.

That running passed off; did it leave a sore in there?

"No, sir; only a little pimple." Then it got well entirely? "Yes, sir." He has a small orifice, and if he had this running and it got well within two or three days, it was probably from the cause I have just suggested, a little leucorrhœal disturbance.

How long was it after the running stopped that you noticed this sore here? "About two weeks."

About two weeks, then, after this, without any after connection, he had, as he says, this sore, and that has continued ever since. The history seems a little mixed, and it often embarrasses rather than helps the surgeon to make out the diagnosis. Now, we have here the history of a little discharge which came on three or four days after connection, which lasted only three or four days, and then within a couple of weeks a sore makes its appearance here. So, we may have this trouble due to syphilitic condition. That is possible. Although the length of time is unusual, yet, in some cases we do have an ulcerative lesion apparently dependent upon the syphilitic virus in cases where the suppurative disposition is very strong. An accumulation of cells in some instances results in their breaking down much sooner than in others. In the great majority of cases there is very slight or scarcely any tendency to the suppurative condition, while in others the infiltration of a comparatively small amount of cells will soon eventuate in their breaking down and becoming a little ulcerative point. When this is the case, the danger of mistaking it for a simple chancroid is very great.

The distinction that we make between a chancroid and a chancre is not an arbitrary, but a positive one. Whatever shape it may have, or whatever condition it may be found in, it must be followed by syphilis, to prove it to be a chancre. This is an absolute necessity. A chancre is not a chancre because it is a little nodule, nor because it has a slight abrasion, nor because it has gone on to ulceration, nor because it is single; but it is a chancre because it is followed by syphilis. These conditions that we associate with it, and which are common to it, lead us in the way of diagnosis; and when we find these conditions we have reason to suppose that we have the initial lesion of syphilis. But if the papule does not go on and enlarge, and implicate the glands at the groin in a peculiar way that the syphilitic trouble implicates them, if it is not followed by any syphilitic lesion, then we know that we have made a mistake; that something has occurred in this case to obscure the diagnosis, to deceive us, to give us the impression that there was a true chancre when none existed. We have to bear in mind that all the characteristics of the initial lesion of syphilis may be present without its being truly an initial lesion of syphilis. So that we have to wait oftentimes until something else has occurred, before we can give an expression of opinion in regard to it.

Now, here we have, apparently. an ulcerative lesion, which is somewhat indurated. This leads us to consider a point of importance in differential diagnosis. In chancroid we have simply ulceration without induration; the tissues are supple and perfectly soft. In the other case we have it characterized by a certain amount of induration, in degree, from that which simply thickens the integument to that which is described as cartilaginous hardness. This is a characteristic mark of the initial lesion of syphilis. And yet a simple sore, a simple chancroid, may, by irritation, become indurated, so that you cannot tell the difference between that and the initial lesion of syphilis; and this must be borne in mind. In this case, as before mentioned, there is a certain amount of induration with it. Now, whether that is the result of some application which may have been made, or whether it is the result of infiltration of cell material, is a question which we cannot at once decide. Here we have a secretion which is not a mucous secretion, which is not a serous secretion, but it is evidently of a more or less purulent character. Again, we must remember that the simple serous secretion of a chancre may be turned into a purulent secretion by irritation, and that this irritation may be the result of applications made to it of caustics, which are used for the purpose of destroying chancroidal sores. It may be the result of friction from clothes, necessarily occurring in walking, or at the patient's business. Any irritation of the initial lesion of syphilis may produce, instead of the characteristic serous secretion, a purulent secretion, which purulent secretion has the same characteristics as the purulent secretion of the chancroid, namely, to set up an ulcerative action upon the mucous membrane, or upon the parts which come in contact with it; so that you may have the initial lesion of syphilis coming on characteristically, which shall never have been mixed up with any other sort of trouble at all; a pure, simple, initial lesion of syphilis, which may go on under these circumstances and produce a purulent secretion characteristic of chancroid. Now, we may find that this condition here, which came on nearly three weeks after connection, is still the initial lesion of syphilis which has been irritated up to a purulent point, and, now, within the last few days, has given rise to chancroids without any further connection. There is nothing more positively known, in regard to the history of this disease, than that the initial lesion of syphilis, irritated, produces a purulent secretion which, inoculated, produces characteristic chancroid. Mr. Boeck, the great apostle of syphilization, he who syphilized a great many individuals and demonstrated this, found that in order to get the material for producing the characteristic lesion by inoculation, in the easiest manner, he had to take the ordinary initial lesion of syphilis and irritate it by the application of savin ointment until it became purulent. Then he took this and inoculated with it until it would inoculate no longer; then he would take a fresh subject, and so on, until each patient would no longer respond to inoculation. I simply speak of this in passing, to show you how well known is this fact, that pus received from an irritated syphilitic sore possesses the property in question.

So, then, although we have these ulcerative lesions, some of which are characteristic of chancroid, and which I present to you as specimens of classical chancroid, yet we are at a loss to know whether this young man has chancroid only, or not. This chancroid here is the leading feature of the case. That this one here is an accidental chancroid we know, but he has another sore there which is at least questionable. Now, if this is truly an initial lesion of syphilis, we shall find that it has gone on in the usual way of initial lesion of syphilis; that the cell proliferation which is characteristic of this lesion has followed the track of the vessels running between this initial lesion and the groin on either side, and an accumulation of these cells has taken place. We examine on either side, and we find on this side no more than an ordinary amount of enlargement; still here is a pretty good-sized gland. There is a little more than the ordinary amount of enlargement here, on the left side, and on the right side there is quite a good-sized gland. Did you ever notice that? "Yes, sir, I noticed it first recently." Has it been tender at all? "Yes, sir, somewhat. It does not hurt me now."

When we brought this case before you it appeared to be a classical chancroid; it may be that we have something more important; we may have a case of syphilis; and that is why I brought the patient before you, to show you how necessary it is to follow up all the clues which present, that we may not be misled to say here is a case of chancroid which we will heal up, and let the patient go on about his business, when, after awhile, you would learn that his trouble was not a chancroid, but syphilis. This mistake happens every day.

We have here painless glandular enlargements in the vicinity of this lesion. There is a peculiar glandular enlargement associated with chancroid which is characteristic; it differs from that of syphilis. The gland enlargement which is characteristic of chancroid is inflammatory in its commencement, inflammatory in its progress, resulting in abscess, I say, almost inevitably. Six months ago I would have said inevitably. I have taught, up to within six months, for many years, that when a gland is enlarged as a result of the chancroid secretion, suppuration is inevitable; a chancroid is at that moment established in the centre of that gland, which goes on inevitably to suppuration and the formation of an abscess, the secretion of which is chancroidal. But the experiments which were made last winter in Blackwell's Island Hospital, under my supervision—the administration of the calcium sulphide in all cases of bubo associated with chancroid—has led to the belief in the possibility of that suppurative action being arrested, because fifteen out of eighteen buboes so associated with presenting chancroids were brought to resolution. Now, we have not had enough experience on that point to state a positive conclusion, but we can say that the administration of a twelfth of a grain of the calcium sulphide every two hours during the day, to these patients, combined with other treatment, such as pressure and iodine, resulted in the resolution of fifteen out of eighteen. I speak of it in passing because I have so long and so often taught to the contrary, that they never can be arrested, that I wish to express here my doubt about it. I do not think we have experience enough yet in regard to the effect of this remedy, but I think there may be a doubt in regard to what I used to teach. At any rate, chancroid is inflammatory in its character, and its tendency is to go on to suppuration, and it will do so unless it is combated by, as far as I know, only one means, that is, that which I have just suggested.

The bubo associated with the initial lesion of syphilis, on the contrary, never suppurates. Absolutely never suppurates, except—and all rules have exceptions—in cases of highly scrofulous nature, or where, becoming very large, they become subjected to irritation by friction, in the business, perhaps, of the individual, or from carelessness in walking, under circumstances which would almost naturally produce inflammation.

Now, here we find glands which are recognized by the patient as of recent enlargement, and are now painless. Recent painless enlargement of the lymphatic glands is evidence, very positive and strong evidence, of the presence of a syphilitic influence. Scrofulous glands enlarge slowly. Enlarged glands which come from ordinary causes, from irritation of the lymphatic vessels, from strain, and from wounds, are painful. They come on quickly, but they are painful. Here, however, they are painless, and the enlargement rapid, which is an important element in the diagnosis.

The next thing we would look for is an eruption. It has been six weeks since he had connection, so that is too soon to look for the next characteristic of the disease which we seem to be following now, a syphiltic trouble, and that is, a roseola eruption. Now, if in connection with this history—and here the element of time, you see, is of value —a sore, which came on nearly three weeks after connection, then followed by a painless enlargement of glands—there should follow a roseola, we should not hesitate any longer to say that the man has syphilis. But now we must wait. The case is mixed, and we must wait for time to clear it up. I do not know of any other point which will be of service to us. The whole trouble is still local. Until we get an enlargement of glands at a distance, or a roseola, we have reason to suppose that the disease is confined to this particular locality, and that he has not, as used to be believed, a disease which pervades the entire economy at once. It is a satisfaction to know this; to be assured that the approaches which it makes to the general system are slow, and they give us an opportunity of meeting the disease and obstructing it in various ways by treatment.

Let us, however, examine him for enlarged glands at a distance. We look for these enlarged glands in the neck and in the epitrochlean regions, preferably. I find an enlarged gland here on the neck, at once. This is sometimes found before we can make out any roseola.

Now, these little chancroids, when they come, are treated in the ordinary way, no matter how they have arisen; whether they have arisen from what is called the true chancroid virus or not. We will have the opportunity of setting this chancroidal virus in its proper place some day. We know it can be produced, certainly, in two ways, and I think it can be produced in a variety of ways. But no matter how it is produced, we strike it with destructive agents which are capable of destroying this peculiar property of active ulceration.—*Medical and Surgical Reporter.*

